# Smooth versus Textured Surfaces: Feature-Based Category Selectivity in Human Visual Cortex

**DOI:** 10.1523/ENEURO.0051-16.2016

**Published:** 2016-09-26

**Authors:** Cesar Echavarria, Shahin Nasr, Roger Tootell

**Affiliations:** 1 Athinioula A. Martinos Center for Biomedical Imaging, Massachusetts General Hospital, Charlestown, Massachusetts 02129; 2 Department of Radiology, Harvard Medical School, Boston, Massachusetts 02115

**Keywords:** FFA, fMRI, functional connectivity, LO, texture

## Abstract

In fMRI studies, human lateral occipital (LO) cortex is thought to respond selectively to images of objects, compared with nonobjects. However, it remains unresolved whether all objects evoke equivalent levels of activity in LO, and, if not, which image features produce stronger activation. Here, we used an unbiased parametric texture model to predict preferred versus nonpreferred stimuli in LO. Observation and psychophysical results showed that predicted preferred stimuli (both objects and nonobjects) had smooth (rather than textured) surfaces. These predictions were confirmed using fMRI, for objects and nonobjects. Similar preferences were also found in the fusiform face area (FFA). Consistent with this: (1) FFA and LO responded more strongly to nonfreckled (smooth) faces, compared with otherwise identical freckled (textured) faces; and (2) strong functional connections were found between LO and FFA. Thus, LO and FFA may be part of an information-processing stream distinguished by feature-based category selectivity (smooth > textured).

## Significance Statement

Preferred stimuli can reveal the processing steps that take place within a given region of the visual cortex. The human lateral occipital (LO) cortex is thought to respond most strongly to images of objects compared with nonobjects. Here, we used an unbiased computational approach to measure simple image features of object and nonobject stimuli, and to generate more specific hypotheses about the optimal stimuli for LO and related visual areas. Using fMRI, we found that cortical visual areas LO and fusiform face area respond selectively to face, object, and nonobject stimuli with smooth surfaces, compared with stimuli with textured surfaces. These findings clarify visual processing steps that are performed within midlevel visual cortex.

## Introduction

One key goal to understanding visual processing has been to determine the preferred stimuli (i.e., those stimuli to which neurons respond most strongly and most selectively) in each visual cortical site. Such stimulus preferences reflect the nature of information processing at each stage of the visual cortical hierarchy. Accordingly, a broad range of techniques, ranging from single-cell electrophysiology to brain imaging, has been applied to characterize the preferred stimulus features in many visual cortical areas.

Defining the preferred stimulus features is especially important in the lateral occipital (LO) region of midlevel human visual cortex. Responses in LO are considered to be “object-selective,” based on a stronger response to the presentation of intact visual objects, compared with various types of nonobject control stimuli (Malach et al., 1995; Kanwisher et al., 1996; Grill-Spector et al., 1998a,b; Kourtzi and Kanwisher, 2000). However, it can be argued that this object-selective characterization of preferred stimuli in LO is ill defined, because there are an unlimited number of possible objects. Thus, a more specific definition of preferred stimuli in LO could help to generate more specific hypotheses about the computations that are executed by neurons in this area.

The definition of LO as object selective also raises further questions about preferred stimuli in this cortical regions. For instance, does LO respond at an equivalently high level to all objects, and at a uniformly lower level to all nonobjects? If not, which specific stimuli are preferred in LO? If preferences exist, would empirically preferred and nonpreferred stimuli scale along an intuitively obvious dimension?

Indirect evidence suggests that at least some components within LO respond preferentially to certain objects compared with others. For instance, multivoxel pattern analysis can decode the category of single objects from the pattern of LO activity (MacEvoy and Epstein, 2007; Cichy et al., 2011). However, the biological mechanisms underlying these pattern classifications remain unclear.

In contrast to early cortical visual areas, higher areas respond selectively to complex stimulus features (Tanaka, 1996; Pasupathy and Connor, 2001), to the point that it becomes difficult to define preferred stimuli using simple methods. Accordingly, many recent studies have defined stimulus preferences in specific areas of higher-level visual cortex based on responses to a common semantic category, including faces (Kanwisher et al., 1997, 1999; Gauthier et al., 2000; Haxby et al., 2000; Grill-Spector et al., 2004; Schwarzlose et al., 2005; Tsao et al., 2006), body parts (Downing et al., 2001; Peelen and Downing 2005), and places (Aguirre et al., 1996; Epstein and Kanwisher, 1998; Hasson et al., 2003; Park and Chun, 2009). However, it remains an open question how (and to what extent) the category selectivity of these areas arises from selectivity to simple visual features, and conjunctions thereof (De Beeck et al., 2008; Rajimehr et al., 2011; Ohayon et al., 2012; Nasr et al., 2014, 2015; Rice et al., 2014).

Here, we used a nonsemantic approach to define preferred stimuli in LO. We hypothesized that the visual features that distinguish objects from nonobjects could be identified and used to make more specific predictions about the preferred stimulus set for LO. To this end, we used a model originally developed for the parametric description and synthesis of visual textures (“texture synthesis”; Portilla and Simoncelli, 2000). This model quantifies a large set of simple features within an image, which we then used to compare images of objects and nonobjects. This approach allowed us to more precisely define preferred stimuli in LO and in potentially coactivated areas [e.g., fusiform face area (FFA); see below].

Our main hypothesis was that this approach would reveal a more specific preferred stimulus set in area LO, compared with the broader semantic category of “objects.” Consistent with this main hypothesis, we found that LO does not respond equivalently to all objects or nonobjects. Moreover, a portion of this response variance reflected stimulus variations in smooth compared with textured surfaces.

A corollary to our main hypothesis is that any stimulus preferences found in LO might also be found in areas with connections to and/or from LO. Consistent with this, we found that the FFA also prefers smooth over textured surfaces, in both face and nonface stimuli. A possible neural link between LO and FFA was supported by the finding of strong functional connections between these two areas, even when subjects were resting with eyes closed.

## Materials and Methods

### Experimental stimuli

#### Visual objects for ranking

A total of 300 images of everyday objects were randomly selected from the BOSS image database (Brodeur et al., 2010). The images were first converted to grayscale format. Then the objects were placed on a gray background, and matched for visual field area, mean luminance, and root mean square (rms) contrast. The objects subtended 18.6° on average (i.e., 714 pixels on our display screen).

#### Synthetic stimuli

Two synthetic stimulus sets were created. First, texture synthetic (TS) stimuli were created by randomly selecting a total of 32 grayscale images of everyday objects on a uniform gray background, not included within the set of 300 images described above. Next, we matched boundary properties among stimuli, as follows. First, functions describing the contour of each of the 32 object stimuli were created by placing a reference point at the center of each object image, then measuring the pixel distance from the reference point to the outermost contour of the object. The process was repeated for all 360° surrounding the reference point, in 1° steps. The result for each object was a one-dimensional function of radius length as a function of direction. The contour function of each object was then Fourier phase scrambled and used to create the contours of 32 different Gaussian noise-filled apertures. Subsequently, the noise was “coerced” to take on the image features of the same object with the use of the parametric texture analysis and synthesis procedure described by Portilla and Simoncelli (2000).

**Figure 1.**
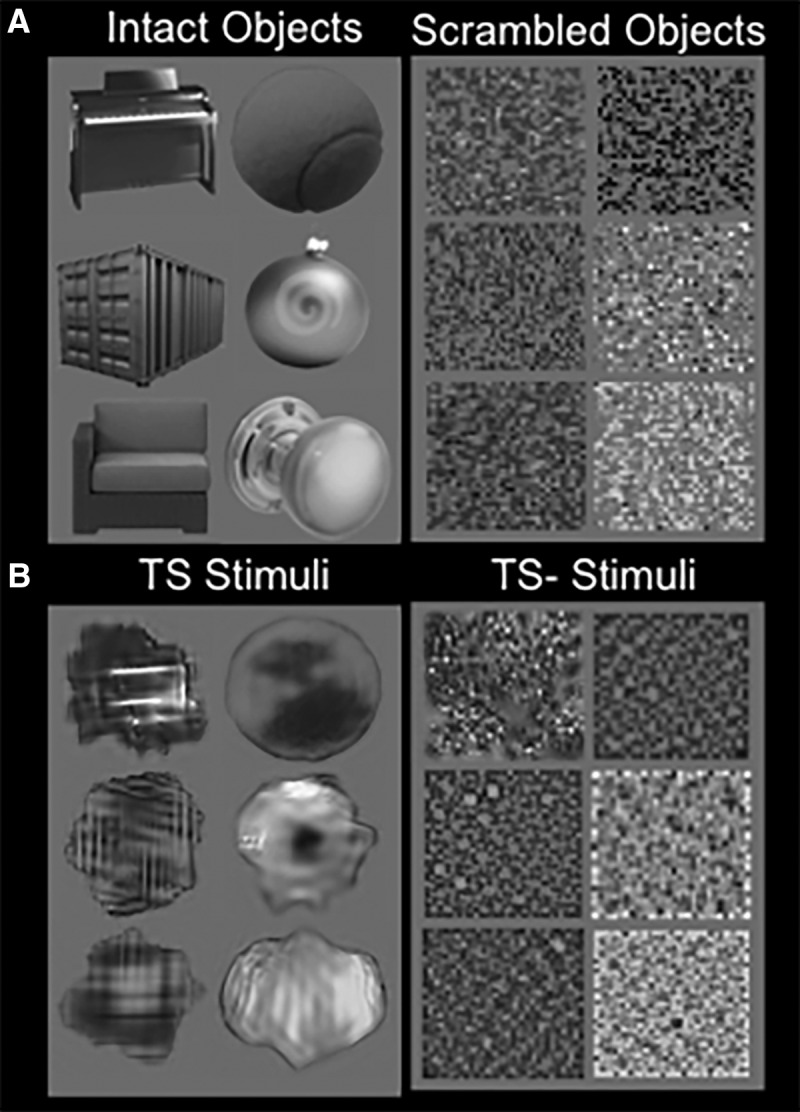
Sample images of objects used to generate synthetic stimuli and resultant stimuli. ***A***, Left, Intact object stimuli used for the imaging and psychophysics experiments (Experiments 1a and 1b), and to generate the TS stimuli. Right, Scrambled object stimuli used to generate the TS− set of stimuli. ***B***, Sample synthetic stimuli used for the imaging and psychophysics experiments (Experiments 1a and 1b). Left, TS stimuli. Right, TS− stimuli.

The second set of synthetic stimuli was termed TS− stimuli. In each of the TS− stimuli, each of the original object stimuli was “grid scrambled” by dividing the image into a 32 × 32 grid then pseudo-randomly shuffling the individual segments. Gaussian noise-filled square apertures were then matched in surface area with each of the 32 object images. The Gaussian noise within each aperture was then coerced to match the image features of each scrambled object. Examples of the resultant and source stimuli are shown in Figure 1.

#### Face stimuli

A total of eight freckled and eight nonfreckled faces were created with FaceGen (Singular Inversion). Real-life photographs were used to create eight different identities from which a nonfreckled and a freckled face were generated. Thus, the two face conditions were matched for the configuration of facial features, as well as viewpoint and lightning conditions. The computer-generated images were converted to grayscale format, and the faces were cropped out with an elliptic aperture and then placed on a uniform gray background. The border of the faces with the background was softened with a Gaussian blur [full-width at half-maximum (FWHM), 0.3°]. Both face image sets were matched for size, rms contrast, and mean luminance. The cropped faces subtended 19.2° on average.

### Ranking objects based on image statistics

Image features from the intact and scrambled object sets used to generate the synthetic stimuli (described above) were used to rank a third set of 300 independent object images. First, image features were computed for each of the 64 images (32 intact objects, 32 scrambled objects), across four orientations and four spatial scales, using the procedure for parametric texture analysis. The features collected from an image describe the following: (1) the distribution of pixel values; (2) the periodicity of pixel values; (3) the energy at different spatial scales and orientations; (4) magnitude correlations across orientation and spatial scales; and (5) phase correlations across spatial scales.

**Figure 2.**
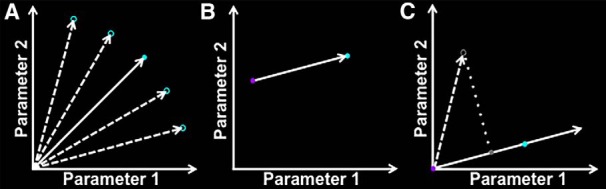
Schematic depiction of the method used to rank 300 objects based on their image statistics. The two-parameter case is depicted for ease of visualization (2457 parameters actually used). ***A***, Image properties of 16 intact objects were measured (unfilled cyan circles). The mean of each parameter was then computed to arrive at the mean “location” for intact objects (filled cyan circle). The same procedure was repeated for the images of scrambled objects (purple circle in ***B***). ***B***, The vector pointing from the scrambled objects to the intact objects in the parameter space was obtained by taking the difference between these two points. ***C***, Subtracting the obtained difference from all points defines a new vector space, with the scrambled objects point at the origin (position = 0). Normalization places the intact objects point at unit length (position = 1). For each of 300 independent objects (unfilled gray circle), image properties were measured and represented as a point in this parameter space. This point was then projected onto the intact–scrambled object axis. The position along this axis denotes the level of similarity between a given object and the scrambled objects image set.

Computing the image features for each image yielded values for the 2457 parameters that the analysis used to describe the image. The mean value for each of these parameters was computed for the intact and scrambled object sets separately (Fig. 2*A*). This yielded two points in the 2457-dimensional parameter space, which were used to define an axis against which to compare an independent set of objects (Fig. 2*B*). This axis was defined such that the point represented by the mean values of the scrambled objects set was at the origin (0), and the point represented by the mean of the intact objects set was at unit length (1).

The image features were then computed for each of the images belonging to the independent set of 300 objects. The vector described by the parameter values for the image was then projected onto the intact–scrambled axis defined above (Fig. 2*C*). Finally, a scaling coefficient was obtained for the projected vector to match its length to the vector describing the intact objects image set. The inverse of this scaling coefficient was used to index the position of the object along the intact–scrambled axis.

**Figure 3.**
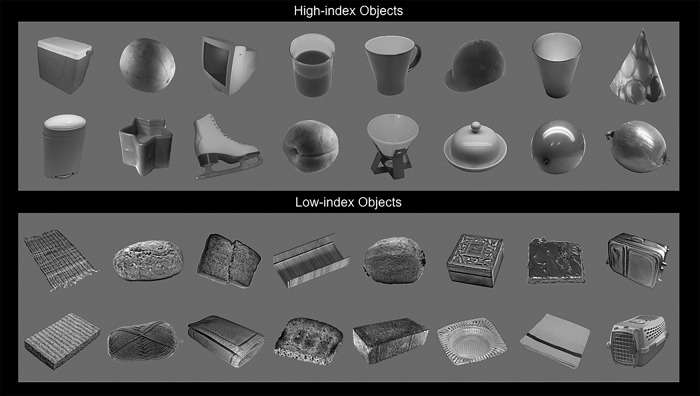
All stimuli from the high-index (smooth) and low-index (textured) stimuli sets used in Experiments 2b and 2c.

**Figure 4.**
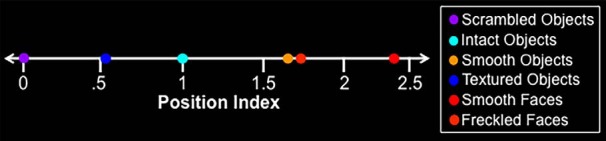
Mean position index for the stimulus sets used in Experiments 1, 2, and 3. Intact objects and scrambled objects sets were used to define the intact–scrambled axis and also served to create the TS and TS− stimuli used in Experiment 1, respectively.

A higher value for this position index indicated a lower similarity in image features to the scrambled object images and, thus, a higher predicted response in LO. Conversely, a lower value indicated a higher similarity to the scrambled object images, based on image features. The 300 objects were ranked with respect to the computed position index. The 16 objects with the highest values were labeled as “high-index” objects. The 16 objects with the lowest values were labeled as “low-index” objects. Both stimulus sets are shown in Figure 3. The mean position indices for all stimulus sets are shown in Figure 4.

### Behavioral procedures

#### Subjects

All subjects participating in the behavioral experiments had normal or corrected-to-normal vision. None of these subjects participated in the imaging experiments. All behavioral and imaging experimental procedures conformed to National Institutes of Health guidelines and protocols were approved by Massachusetts General Hospital. Written informed consent was obtained from all subjects.

#### Stimulus presentation

Stimuli were presented on a 13 inch LCD screen. MATLAB R2013a and Psychophysics Toolbox (Brainard, 1997) were used to control stimulus presentation. Throughout the experiment, subjects kept their head on a chin rest located at a fixed position, thus controlling viewing distance.

#### Visual texture classification

A total of five subjects participated in this psychophysical experiment. Each subject completed a total of 192 trials. Subjects viewed each of the 32 object stimuli a total of six times during the course of the experiment. Each trial began with a period of 500 ms without stimulus presentation in order to allow subjects to fixate a red fixation spot subtending 0.16° at the center of the screen. The initial fixation period was followed by a 1 s presentation of either a high-index or low-index object. This was followed by presentation of a white-noise mask in place of the object stimulus. Subjects were instructed to classify the object presented as visually “smooth” or “textured.” Subjects gave their answer by pressing one of two keys on a keyboard. The ensuing trial began after the subject’s response.

#### Object recognition of high- and low-index objects

A total of five subjects participated in this psychophysical experiment. To avoid ceiling effects in recognition performance, each object stimulus was reduced in retinal size, to subtend 4.65°, and was presented at 10% contrast. Each subject completed a total of 128 trials. Subjects viewed each of the 32 object stimuli a total of four times during the course of the experiment. Each trial began with a period of 500 ms without stimulus presentation to allow subjects to begin fixation on a red fixation spot, which subtended 0.16° at the center of the screen. The initial fixation period was followed by a 50 ms presentation of one of the high-index or low-index objects. This was followed by presentation of a white noise mask in place of the object stimulus along with four possible labels for the presented object. The four options included the appropriate object label (e.g., “apple”) along with three distractors, which were randomly selected from a bank of 99 other object labels. Distractor labels included (but were not limited to) labels for the other 31 objects in the stimulus bank. Subjects were instructed to choose the most accurate label for the object presented during the trial. Subjects gave their response by pressing one of four keys on a keyboard.

#### Object recognition for synthetic stimuli

Five subjects participated in this psychophysical experiment. Each subject completed a total of 480 trials. Subjects viewed each of the 96 stimuli (32 stimuli/condition; three conditions) a total of five times during the course of the experiment. Each trial began with a period of 500 ms with no stimulus presented, during which subjects began fixating a red central spot subtending 0.16°. The initial fixation period was followed by a 1 s presentation of one of the intact objects (TS stimuli) or scrambled objects (TS− stimuli). Finally, a white noise mask appeared in place of the stimulus, along with four possible labels for the presented stimulus. The four options included the appropriate label (e.g., “rug”) along with three distractors randomly selected from a bank of 99 other object labels. Distractor labels included (but were not limited to) labels for the other 31 objects in the stimulus bank. Subjects were instructed to choose the most appropriate label for the stimulus presented during the trial. Subjects gave their response by pressing one of four keys on a keyboard. All trials with intact objects were presented at the beginning of the experiment in order to prevent subjects from inferring the identity of synthetic stimuli.

### Imaging

#### Subjects

For each experiment, subjects were selected randomly from a pool of 30 subjects (12 females; age range, 20–38 years). All statistical analyses are summarized in Table 1. Table 2 indicates which subjects participated in each experiment. All subjects had normal or corrected-to-normal visual acuity and radiologically normal brains, without a history of neuropsychological disorder.

**Table 1:** Summary of key statistical analyses

	Data structure	Test type	Observed power
a	Normal distribution	One-sample *t* test	0.15
b	Normal distribution	One-sample *t* test	0.10
c	Normal distribution	Paired-sample *t* test	0.05
d	Normal distribution	One-sample *t* test	1.0
e	Normal distribution	Paired-sample *t* test	0.96
f	Normal distribution	Paired-sample *t* test	0.54
g	Normal distribution	Paired-sample *t* test	0.99
h	Normal distribution	Paired-sample *t* test	0.66
i	Normal distribution	Paired-sample *t* test	0.21
j	Normal distribution	Paired-sample *t* test	1.0
k	Normal distribution	Paired-sample *t* test	0.10
l	Normal distribution	Paired-sample *t* test	0.62
m	Normal distribution	Paired-sample *t* test	0.23
n	Normal distribution	Paired-sample *t* test	1.0
o	Normal distribution	Paired-sample *t* test	1.0
p	Normal distribution	Paired-sample *t* test	0.85
q	Normal distribution	Two-sample *t* test	1.0
r	Normal distribution	Paired-sample *t* test	0.56
s	Normal distribution	Paired-sample *t* test	0.50
t	Normal distribution	Paired-sample *t* test	1.0
u	Normal distribution	Paired-sample *t* test	0.95
v	Normal distribution	Paired-sample *t* test	0.59
w	Normal distribution	Paired-sample *t* test	0.11
x	Normal distribution	Paired-sample *t* test	0.12
y	Normal distribution	Paired-sample *t* test	0.08
z	Normal distribution	Paired-sample *t* test	0.88
aa	Normal distribution	Paired-sample *t* test	0.20
ab	Normal distribution	Paired-sample *t* test	0.74

**Table 2:** Tabulation of subject participation for all imaging experiments

Subject ID	Experiment 1c	Experiment 2c	Experiment 3b	Experiment 4
1	X	X	X	X
2			X	
3	X		X	X
4	X	X	X	X
5			X	X
6	X		X	X
7	X	X	X	X
8	X		X	X
9		X	X	X
10		X	X	X
11	X	X	X	X
12	X	X	X	X
13		X		
14	X	X	X	X
15		X		X
16			X	X
17		X		X
18		X	X	X
19				X
20				X
21				X
22				X
23				X
24				X
25				X
26				X
27				X
28				X
29				X
30				X
31				X

#### Experiment 1: original and synthetic stimuli

A total of 10 subjects (7 females) participated in this experiment. In each session, stimuli were presented in blocks of intact objects (TS stimuli) or scrambled objects (TS− stimuli; 16 images/block; duration, 1 s/image). Each subject participated in six runs, and each run included six blocks.

#### Experiment 2: high- and low-index objects

A total of 12 subjects (7 females) participated in this experiment. In each session, stimuli were presented in blocks of either high-index or low-index objects (16 images/block; duration, 1 s/image). Each subject participated in six runs, and each run included four blocks.

#### Experiment 3: freckled and nonfreckled faces

A total of 15 subjects (8 females) participated in this experiment. In each session, stimuli were presented in blocks of either nonfreckled or freckled faces (16 images/block; duration, 1 s/image). Each subject participated in six runs, and each run included four blocks.

#### Functional connectivity

A single functional run lasting 6.2 min (124 time points) was acquired from each of 29 subjects (12 females). Subjects were instructed to fixate the center of a blank screen for the duration of the run with no other task. This run was used for subsequent functional connectivity analysis.

#### Stimulus presentation and task

Stimuli were presented via LCD projector (XG-P25, Sharp; 1024 × 768 pixels, 60 Hz refresh rate) onto a rear-projection screen. MATLAB R2013a (MathWorks) and Psychophysics Toolbox (Pelli, 1997; Brainard, 1997) were used to control stimulus presentation.

Each run began and ended with an additional fixation-only block (16 s). All images were centered on the display screen against a spatially uniform gray background. In all experiments, subjects were instructed to maintain fixation on a very small (0.1°) central red square, during performance of a dot detection task using the button box in the scanner. The probe dot appeared at unpredictable times (100 ms random shift from each stimulus onset), distributed randomly across the display with equal spatial probability. The detectability of the probe dot was manipulated by varying its cyan/white ratio (decreased saturation = decreased detection). Threshold was modulated by the staircase method, converging on 75% correct. To reduce response variability, the dot size varied with eccentricity.

#### Data acquisition

All subjects were scanned in a horizontal 3 T scanner (Magnetom Trio Tim System, Siemens). Gradient EPI sequences were used to acquire functional images (TR, 2000 ms; TE, 30 ms; flip angle, 90°; 3.0 mm isotropic voxels; and 33 axial slices). For functional connectivity scans, each TR was 3000 ms. Forty-seven axial slices were acquired in each TR. In the fMRI scans, the field of view included the whole brain, for all subjects. A 3D MP-RAGE sequence (1.0 mm isotropic) was also used for high-resolution anatomical imaging from the same subjects. Functional and anatomical data were preprocessed and analyzed using FreeSurfer and FS-FAST (version 5.3; http://surfer.nmr.mgh.harvard.edu/; Fischl, 2012).

#### Localizers

To localize scene-selective and face-selective areas, subjects were presented with an independent set of stimuli, contrasting images of real-world faces with scenes. Scene stimuli consisted of various full-screen images of outdoor and indoor scenes. Face stimuli consisted of full-screen mosaics that included different equal-sized faces adjacent to each other. All stimuli spanned the whole screen, which allowed both conditions to be retinotopically matched. Subjects viewed these stimuli in the scanner while performing a task detecting color changes on a small fixation dot at the center subtending 0.1°. Stimuli were blocked into intact and scrambled conditions (16 images/block; duration, 1 s/image). Each subject participated in six runs, and each run included eight blocks.

To retinotopically define early visual areas, subjects viewed an independent set of stimuli consisting of wedge-shaped apertures extending from the center to the edge of the screen, which contained images of faces or objects. The wedges were located (1) on either side of the center across the horizontal meridian of the screen, (2) on the upper half of the screen along the vertical meridian, or (3) on the lower half of the screen along the vertical meridian. Subjects viewed these stimuli in the scanner while performing a task detecting color changes on a small fixation dot at the center subtending 0.1°. Stimuli were blocked into the described three conditions (16 images/block; duration, 1 s/image). Each subject participated in six runs, and each run included six blocks.

**Figure 5.**
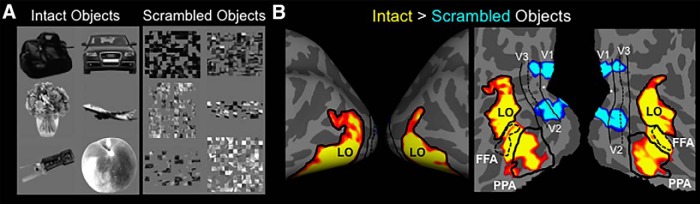
LO localizer stimuli and results for subjects in Experiment 1. ***A***, Examples of stimuli used to localize LO for all subjects. Left, Intact object stimuli. Right, Grid-scrambled object stimuli. ***B***, Left, Lateral view of both hemispheres of a group average activity map, based on fixed-effect analysis, in surface-inflated format showing the response to intact vs scrambled objects for 10 subjects. Right, The same group average map in a flattened surface format. Absolute threshold range. 10^−16^ to 10^−32^. Borders of LO and other regions of interest are delineated with black lines. The asterisk marks the location of the foveal representation. Light gray denotes gyri, and dark gray denotes sulci.

To localize object-selective areas, subjects viewed an independent set of stimuli contrasting images of intact versus scrambled real-world objects (Fig. 5*A*). The intact object stimuli consisted of 37 images of everyday objects on a uniform gray background. The grid-scrambled object stimuli were generated by dividing each object image into a 16 × 16 grid and pseudo-randomly shuffling the individual segments. None of these stimuli were used in any of our main experiments. Subjects viewed these stimuli in the scanner while performing an unrelated (“dummy”) task, detecting color changes on a small fixation dot at the center subtending 0.1°. Stimuli were blocked into intact and scrambled conditions (16 images/block; duration, 1 s/image). Each subject participated in six runs, and each run included eight blocks.

#### Data analysis

For each subject, we reconstructed the cortical surface based on the high-resolution anatomical data (Fischl et al., 1999). All functional images were corrected for motion artifact and then spatially smoothed using a 3D Gaussian kernel with 2.5 mm FWHM, and normalized across scans. For functional connectivity analyses, a Gaussian kernel with 6 mm FWHM was used. To estimate the intensity of the hemodynamic response, a model based on a γ function was fit to the fMRI signal, and then the average signal intensity maps were calculated for each condition (Friston et al., 1999).

For data used in the functional connectivity analysis, several sources of spurious variance were removed by finding and then removing the first principal component of the following nuisance variables: (1) the signal obtained from all voxels in CSF; (2) the signal obtained from all voxels in the white matter; and (3) the signal obtained from averaging across all voxels (global signal). The time course of the seed region described below was then correlated against all voxels of the brain. Finally, correlation values were projected onto the inflated/flattened cortex after a rigid coregistration of functional and anatomical volumes.

For all other runs, voxel-wise statistical tests were conducted by computing contrasts based on a univariate general linear model. The significance levels were then projected onto the inflated/flattened cortex after a rigid coregistration of functional and anatomical volumes. To generate group-averaged maps, functional maps were spatially normalized across sessions and across subjects using Freesurfer. Next, activity within each individual’s brain was spatially transformed onto the averaged human brain using a spherical transformation (Fischl et al., 1999), and then averaged using both fixed and random-effects models (Friston et al., 1999).

#### Region of interest analyses

For each subject, regions of interest (ROIs) were defined for the object-selective area LO, the face-selective area FFA, and the scene-selective area parahippocamapal place area (PPA) based on independent localizing stimuli (see above). The posterior fusiform sulcus (pFS) defined the border between adjacent visual areas LO and FFA (i.e., the ROI for LO extended to, and included, the pFS). Early visual areas V1, V2, and V3 were defined by contrasting the activity maxima to horizontal and vertical meridian stimuli, as described above. The midpoint of the responses to horizontal and vertical meridians defined the borders between adjacent early visual areas.

In all analyses, fMRI activity for each condition was measured relative to the activity during presentation of a uniform gray stimulus (baseline). Because group-averaged activity maps showed that all reported effects were generated bilaterally (without any apparent difference between left and right hemispheres; Tables 3, 4, 5), activity from both hemispheres was averaged in all ROI analyses to strengthen the power of all statistical tests. Paired-sample *t* tests were used to compare individual conditions. A selectivity index was computed for all stimulus contrasts in order to better visualize the magnitude of the contrast. The selectivity index for condition A over condition B was computed using the percentage signal change for each condition, as follows: (condition A − condition B)/(condition A + condition B). Observed power was calculated *post hoc* with G*Power 3.1 (Faul et al., 2007).

**Table 3:** Percentage signal change values by hemisphere for Experiment 1c ±SD

	Left hemisphere	Right hemisphere
V1		
Intact	1.37 ± 0.13	1.37 ± 0.13
TS	1.50 ± 0.12	1.44 ± 0.12
TS−	2.13 ± 0.13	2.01 ± 0.18
V2		
Intact	1.24 ± 0.10	1.31 ± 0.12
TS	1.27 ± 0.08	1.31 ± 0.12
TS−	1.56 ± 0.10	1.61 ± 0.16
V3		
Intact	1.34 ± 0.15	1.34 ± 0.14
TS	1.25 ± 0.11	1.23 ± 0.10
TS−	1.38 ± 0.08	1.34 ± 0.10
LOC		
Intact	0.83 ± 0.08	0.93 ± 0.10
TS	0.56 ± 0.05	0.67 ± 0.07
TS−	0.34 ± 0.04	0.48 ± 0.06
FFA		
Intact	0.77 ± 0.09	0.77 ± 0.09
TS	0.54 ± 0.06	0.57 ± 0.08
TS−	0.41 ± 0.04	0.49 ± 0.12

**Table 4:** Percentage signal change values by hemisphere for Experiment 2c ±SD

	Left hemisphere	Right hemisphere
V1		
Smooth objects	0.92 ± 0.09	0.82 ± 0.09
Textured objects	1.63 ± 0.11	1.50 ± 0.13
V2		
Smooth objects	0.92 ± 0.06	0.88 ± 0.06
Textured objects	1.33 ± 0.07	1.28 ± 0.09
V3		
Smooth objects	0.98 ± 0.09	0.92 ± 0.06
Textured objects	1.26 ± 0.11	1.18 ± 0.09
LOC		
Smooth objects	0.99 ± 0.09	1.00 ± 0.08
Textured objects	0.81 ± 0.09	0.82 ± 0.06
FFA		
Smooth objects	0.96 ± 0.17	1.10 ± 0.10
Textured objects	0.79 ± 0.19	1.05 ± 0.15

**Table 5:** Percentage signal change values by hemisphere for Experiment 3b ±SD

	Left hemisphere	Right hemisphere
V1		
Smooth faces	1.08 ± 0.05	1.03 ± 0.06
Freckled faces	1.72 ± 0.04	1.62 ± 0.04
V2		
Smooth faces	1.02 ± 0.03	0.99 ± 0.03
Freckled faces	1.35 ± 0.03	1.26 ± 0.03
V3		
Smooth faces	0.94 ± 0.03	0.92 ± 0.02
Freckled faces	1.10 ± 0.03	1.05 ± 0.03
LOC		
Smooth faces	0.71 ± 0.01	0.91 ± 0.02
Freckled faces	0.61 ± 0.02	0.79 ± 0.03
FFA		
Smooth faces	0.89 ± 0.02	1.08 ± 0.02
Freckled faces	0.78 ± 0.04	0.95 ± 0.03

#### ROIs for functional connectivity analysis

Three topographically circular ROIs (radius, 6 mm) were defined within each hemisphere in a common cortical surface space. These ROIs were contained within either FFA, LO, or early visual cortex. More specifically, the ROI contained within FFA was centered on the vertex, which showed the maximum value for the face-versus-place contrast, averaged across all 29 subjects. The ROI for LO was centered on the vertex, with the maximum value for the intact versus scrambled object contrast averaged across all 29 subjects. The ROI for early visual cortex was centered on a location in V3 that was equidistant to the ROIs in FFA and LO. All three ROIs on the common surface were then transformed to each individual’s own surface, independently for each hemisphere. Defining these ROIs in a common surface space provided a standard that best matched the cortical distances across subjects.

Three analyses were performed in each hemisphere to measure functional connectivity. For each analysis, one of the three ROIs served as a seed, while the other two served as sampling ROIs. For each subject, correlation maps were computed by correlating the time course of the seed region with all other voxels in the brain. Correlation values were then averaged across each of the two sampling ROIs and then averaged across hemispheres. The mean correlation value for each ROI was then averaged across subjects. *Post hoc t* tests were used to test for significant differences.

## Results

As a prerequisite for the main experiments, we first localized LO in each of 10 human subjects using a conventional localizer based on fMRI measurements during visual presentation of isolated intact objects (“preferred” stimuli), compared with grid-scrambled versions of the same object set (“nonpreferred” stimuli; Fig. 5*A*). Consistent with many previous results, the intact objects produced higher activity in LO, plus several neighboring visual cortical areas, including FFA and PPA (Fig. 5*B*). Also, as expected, the reverse preference (larger responses to scrambled objects) was found in lower-tier visual cortical areas, including V1, V2, and V3.

### Experiment 1: synthetic stimuli

The following three goals were included in this experiment: (1) to generate unfamiliar, nonobject stimuli that have low-level features matched to either intact or scrambled objects; (2) to confirm that such stimuli were not identifiable in terms of the original objects, based on psychophysics; and (3) using fMRI to test whether such unfamiliar stimuli can evoke cortical activity differences analogous to those evoked by the standard localizing stimulus contrast (i.e., intact vs scrambled objects).

### Experiment 1a: stimulus synthesis

We used a parametric texture analysis and synthesis method (see Materials and Methods; Portilla and Simoncelli, 2000) to generate unfamiliar nonobject stimuli. This unbiased method measures a set of simple image features within a “seed” image (e.g., one of our intact or scrambled objects) and generates a new image with equivalent image features. The images of intact and scrambled objects used for stimuli generation were independent of those used to localize LO above (Fig. 1*A*, seed images). The following two sets of stimulus images were generated: (1) TS stimuli based on the intact objects; and (2) an analogous set of stimuli (TS−) based on the scrambled objects. Thus, these TS and TS− stimuli had low-level features that were matched to the original intact and scrambled objects, respectively. Figure 1*B* shows examples of such stimuli.

### Experiment 1b: identification of synthetic stimuli

Both sets of synthetic stimuli were designed to be unfamiliar (i.e., not identifiable relative to the familiar objects from which each synthesized image was derived). To confirm that all the synthesized images (especially those derived from intact objects) were unidentifiable, five naive subjects were presented sequentially with each member of the stimulus set [intact object (TS) or scrambled object (TS−)] and were asked to select the identity of the original object from four possible labels (e.g., “mug”; see Materials and Methods). In each trial, one of the labels accurately described the original object, and the remaining three labels were randomly selected from a bank of 99 other object labels.

**Figure 6.**
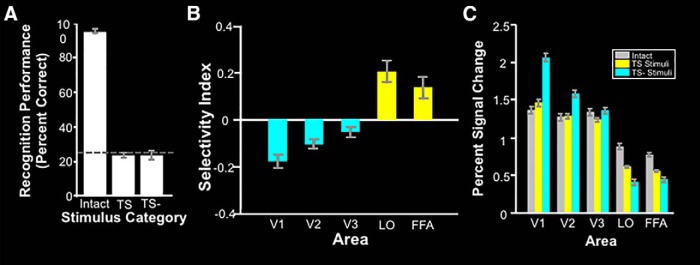
Behavioral and imaging results for Experiment 1. ***A***, Mean recognition performance ± SEM for five subjects in a four-alternative forced-choice task. Dashed line indicates chance performance (25%). ***B***, ROI results from the same group of subjects shown in Figure 1*B* (*n* = 10) shown as the mean of the selectivity index ± SEM for the TS over the TS− condition. ***C***, Percentage signal change from baseline for intact objects, TS stimuli, and TS− stimuli separately. Blue bars indicate ROIs for which the response to TS stimuli is significantly lower than the response to TS− stimuli. Yellow bars indicate the opposite effect. See Table 3 for raw values for each hemisphere.

A one-sample sample *t* test showed that recognition performance did not differ significantly from chance level (25%) for both TS^a^ (*t*_(4)_ = −1.19; *p* = 0.30) and TS− stimuli^b^ (*t*_(4)_ = −0.85; *p* = 0.44). Moreover, we found no significant difference in recognition performance between the TS and TS− stimuli^c^ (*t*_(4)_ = 0.31; *p* = 0.77). As an expected control result, subjects were able to match the intact objects with their corresponding label, well above chance^d^ (93 ± 1.05% correct; *t*_(4)_ = 65.14; *p* < 10^−6^; Fig. 6*A*). On this basis, both the TS and TS− stimuli may be considered to be unfamiliar nonobjects.

### Experiment 1c: fMRI responses to synthetic stimuli

Next, we used fMRI to test whether TS and TS− stimuli produced activity differences analogous to those produced by the standard LO localizer (i.e., whether the LO response was greater to TS compared with TS− stimuli; *n* = 10). Such an outcome would support our hypothesis that the low-level properties isolated by the synthetic stimuli influence LO responses in a general way, potentially in response to any visual stimulus.

As hypothesized, results of an ROI analysis (see Materials and Methods) showed that TS stimuli evoked significantly higher activity than TS− stimuli in independently localized LO^e^ (*t*_(9)_ = 4.68; *p* < 0.01). Specifically, TS− stimuli evoked a response that was 33% weaker, compared with TS stimuli. A higher response to TS stimuli was also found in FFA^f^ (*t*_(9)_ = 2.81; *p* = 0.02), a result that suggests a link between LO and FFA. Conversely, TS− stimuli evoked significantly more activity in the lower-tier cortical visual areas V1^g^ (*t*_(9)_ = −6.45; *p* < 10^−3^), V2^h^ (*t*_(9)_ = −4.56; *p* < 0.01), and V3^i^ (*t*_(9)_ = −2.50; *p* = 0.03). This response profile (i.e., a relatively higher response to TS stimuli in LO and FFA, and a conversely higher response to TS− stimuli in lower-level areas) is analogous to the response profile produced by the conventional LO localizer contrasting the response to intact objects with that to scrambled objects in this group of subjects (Fig. 6*B*). Thus, these results suggest that specific low-level image properties do contribute to LO activity, independent of possible influences related to recognition.

### Experiment 2: smooth versus textured objects

The above results suggest that the low-level visual features isolated by the TS versus TS− stimuli modulate LO responses to unfamiliar nonobjects. In Experiment 2, we tested whether these low-level features would also modulate responses to familiar objects. This experiment used a three-stage design similar to that used in Experiment 1 (stimulus set generation, psychophysics, and fMRI).

### Experiment 2a: stimulus selection for object preference test

Experiment 2a used the texture synthesis model to measure simple image features of 300 images of everyday objects (see Materials and Methods). For each object, the measured image features were compared with the image features of the intact and scrambled objects used to generate the synthetic stimuli in Experiment 1. Specifically, we computed an index for the position of the object along the axis defined by the intact and scrambled objects in the 2457-dimensional parameter space used to describe the images (Fig. 2). For each object, a higher index indicated a higher predicted response in LO, and vice versa.

Based on this scale, we selected the 16 objects with the highest indices, and the 16 objects with the lowest indices, referred to as high-index and low-index objects, respectively. Figure 3 shows both stimulus sets.

### Experiment 2b: identification of smooth versus textured objects

**Figure 7.**
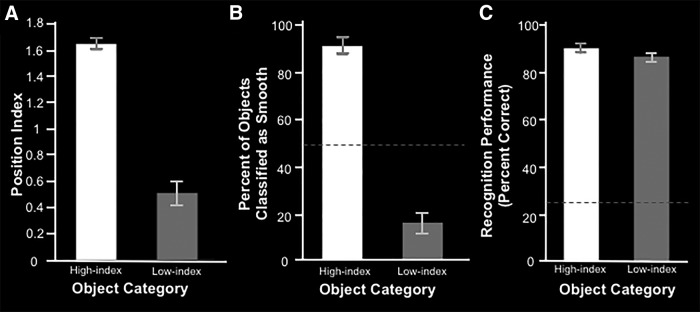
Computational results for Experiment 2a and behavioral results for Experiment 2b. ***A***, Mean position index ± SEM for the top 16 (high-index/smooth) and bottom 16 (low-index/textured) objects. ***B***, Visual appearance classification performance ± SEM for five subjects in a two alternative forced-choice task (smooth or textured). Data are shown as the mean percentage of trials in which subjects classified the objects as smooth. The dashed line indicates chance performance (50%). ***C***, Mean recognition performance ± SEM for five subjects in a four-alternative forced-choice task. Dashed line indicates chance performance (25%).

By observation, it appeared that the high-index objects had smoother surfaces, compared with the low-index objects; which appeared to have more textured surfaces. To quantify this impression, we conducted a psychophysical experiment outside the scanner. In each trial, each of five naive subjects were presented with an exemplar from either the high-index or low-index object sets (i.e., based on position index values) and were asked to classify the surface of that object as either smooth or textured (see Materials and Methods). A paired *t* test showed that the high-index objects were classified as smooth by the subjects significantly more often than the low-index objects^j^ (*t*_(4)_ = 13.35; *p* < 10^−3^; Fig. 7*B*). Based on this finding, and for brevity, below we refer to the high-index objects as smooth, and the low-index objects as textured.

An additional control experiment confirmed that subjects recognized smooth objects as readily as the textured objects. Another group of five naive subjects were presented with an exemplar from either of the two object stimuli sets and were asked to select the identity of the objects from four possible labels (e.g., “balloon”). A paired *t* test showed no significant difference in recognition performance between smooth and textured objects^k^ (*t*_(4)_ = 0.96; *p* = 0.39; Fig. 7*C*).

### Experiment 2c: fMRI responses to smooth versus textured objects

**Figure 8.**
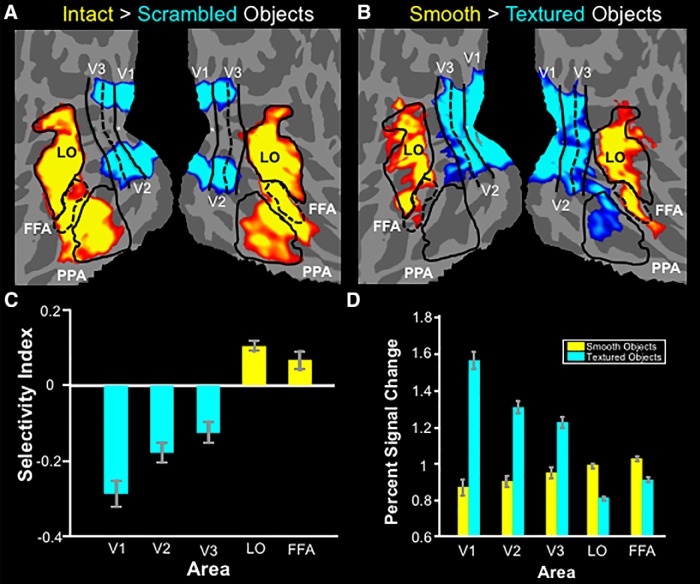
fMRI results from Experiment 2c. ***A***, Flattened-surface format for both hemispheres of a group average map, based on fixed effect analysis showing the response to intact versus scrambled objects for 12 subjects. Absolute threshold range: 10^−30^ – 10^−50^. Other conventions are as in Figure 1*B*. ***B***, Flattened-surface format for both hemispheres of a group average map, based on random-effects analysis, showing the response to smooth vs textured (high-index vs low-index) objects for the same 12 subjects. Absolute threshold range, 0.05 to 10^−3^. ***C***, ROI results for the same 12 subjects shown as the mean of the selectivity index ± SEM for the smooth over textured objects. ***D***, Percentage signal change from baseline for smooth and textured objects, separately. Blue bars indicate ROIs for which the response to smooth objects is significantly lower than the response to textured objects. Yellow bars indicate the opposite preference. Error bars indicate SEM. See Table 4 for raw values for each hemisphere.

Our main hypothesis, plus the results from Experiment 2a, predicted that smooth objects would activate LO more than textured objects. To test this prediction, we conducted an fMRI experiment in which the smooth and textured objects were presented in separate conditions (*n* = 12). The object stimuli used here were identical to those tested in the psychophysical experiment (Experiment 2b). A conventional localizer was used to independently define LO for each of the 12 participating subjects (Fig. 8*A*).

As predicted, LO showed higher activity in response to smooth (rather than textured) objects in the group-averaged map generated based on a random-effects analysis (Fig. 8*B*). A subsequent ROI analysis (Fig. 8*C*,*D*) confirmed that smooth objects evoked significantly higher activity in LO^l^ (*t*_(11)_ = 8.61; *p* < 10^−5^). Specifically, textured objects evoked a response that was 18% weaker, compared with smooth objects. As in Experiment 1, this higher response to smooth objects was also found in FFA^m^ (*t*_(11)_ = 4.08; *p* < 0.01). The opposite bias was found in lower-tier retinotopic areas, as follows: textured objects evoked higher activity in V1^n^ (*t*_(11)_ = −7.89; *p* < 10^−5^), V2^p^ (*t*_(11)_ = −6.37; *p* < 10^−4^), and V3 ^o^(*t*_(11)_ = −4.49; *p* < 10^−3^). Thus, in both the group map and the ROI results, the distribution of the smooth versus textured preferences was similar to that found in the standard localizer for LO, for the same group of subjects.

These results confirmed that LO does not respond uniformly to all objects. Instead, it responds better to objects with smooth surfaces, compared with textured surfaces.

### Experiment 3: smooth versus freckled faces

As described above, a corollary to our hypothesis was that any stimulus preference in LO might also be reflected in additional visual areas with a neural link to LO. The results from Experiments 1 and 2, plus the localizer results for LO, all showed response covariation in LO and FFA, which suggests a link between these two regions. Thus, in Experiment 3 we tested for an additional response covariation within these two areas, based on stimuli optimized for FFA instead of LO. Given the well accepted selectivity for face stimuli in FFA (Kanwisher et al., 1997; Grill-Spector et al., 2004), we presented two sets of faces that differed only in terms of smooth versus textured surface features. If the selectivity for smooth surfaces is common to both LO and FFA, then a preference for faces with smooth (relative to more textured) complexions might exist in FFA, in addition to LO.

### Experiment 3a: face stimuli

**Figure 9.**
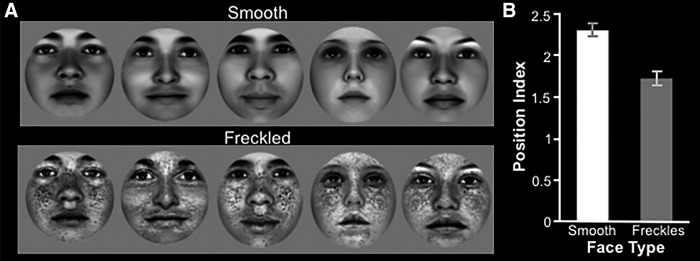
Stimuli from Experiment 3. ***A***, Sample stimuli used in Experiment 3. Top, Computer-generated faces with smooth complexion. Bottom, Same computer-generated faces with freckles. ***B***, Mean position index ± SEM for the smooth and freckled faces used in Experiment 3.

Two sets of computer-generated faces were created to test this idea (see Materials and Methods). One set of faces had a smooth complexion, and the other set was created with more texture by adding freckles to otherwise identical faces (Fig. 9*A*). The use of computer-generated faces made it possible to eliminate many otherwise uncontrolled variables between the two face sets, including viewpoint, lighting, configuration of facial features, and shading. For all stimuli, indices were computed as described in Experiment 2a, so that faces could be objectively assigned to either smooth or textured categories. A two-sample *t* test confirmed that the smooth faces had a significantly higher position index, and thus were quantitatively smoother compared with the freckled faces^q^ (*t*_(14)_ = 8.79; *p* < 10^−6^; Fig. 9*B*).

### Experiment 3b: fMRI responses to smooth versus freckled faces

**Figure 10.**
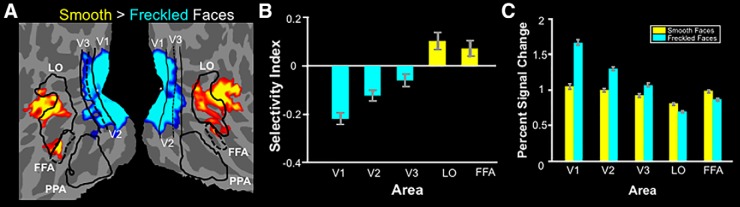
Results from Experiment 3. ***A***, Flattened-surface format of both hemispheres of a group average map, based on random-effects analysis, showing the response to smooth vs freckled faces for 15 subjects. Absolute threshold range, 0.05 to 10^−3^. Other conventions are as in Figure 1*B*. ***B***, ROI results for the same 15 subjects shown as the mean of the selectivity index ± SEM for the smooth over freckled faces. ***C***, Percentage signal change from baseline for smooth and freckled faces, separately. Blue bars indicate ROIs for which the response to smooth faces is significantly lower than the response to freckled faces. Yellow bars indicate the opposite effect. See Table 5 for raw values for each hemisphere.

These face stimuli were then presented in a subsequent fMRI experiment to test the prediction of a higher response in LO and FFA to smooth versus freckled faces (*n* = 15). As predicted, LO and FFA showed higher activity in response to smooth (rather than freckled) faces in the group-averaged map, based on a random-effects analysis (Fig. 10*A*). A subsequent ROI analysis (Fig. 10*B*,*C*) confirmed that smooth faces evoked significantly higher activity compared with freckled faces in both LO^r^ (*t*_(14)_ = 3.06; *p* < 0.01) and FFA^s^ (*t*_(14)_ = 3.07; *p* < 0.01). Conversely, freckled faces evoked higher activity in early visual areas V1^t^ (*t*_(14)_ = −8.51;*p* < 10^−6^), V2^u^ (*t*_(14)_ = −6.02; *p* < 10^−4^), and V3^v^ (*t*_(14)_ = −3.16; *p* < 0.01). Thus, as in Experiments 1 and 2, the response bias in LO and FFA was inverse to that found in lower-tier areas. Consequently, the preference for smooth surfaces in LO and FFA did not appear to reflect a passive transmission of a stimulus bias that arises in earlier visual areas; instead, it was a reversal of that activity bias.

In contrast to LO and FFA, the PPA did not show a consistent bias for either smooth or textured stimuli in the above experiments. Specifically, PPA showed moderately higher activity in response to TS stimuli, compared with TS− stimuli^w^ (*p* < 0.05; *t*_(10)_ = 3.01), higher activity in response to textured objects, compared with smooth objects^x^ (*p* < 0.01; *t*_(11)_ = −3.19), and an equivalent response to smooth and freckled faces^y^ (*p* = 0.62; *t*_(11)_ = 0.52). Based on these inconsistencies, we did not consider the role of PPA further in this study.

### Experiment 4: functional connections between LO and FFA

**Figure 11.**
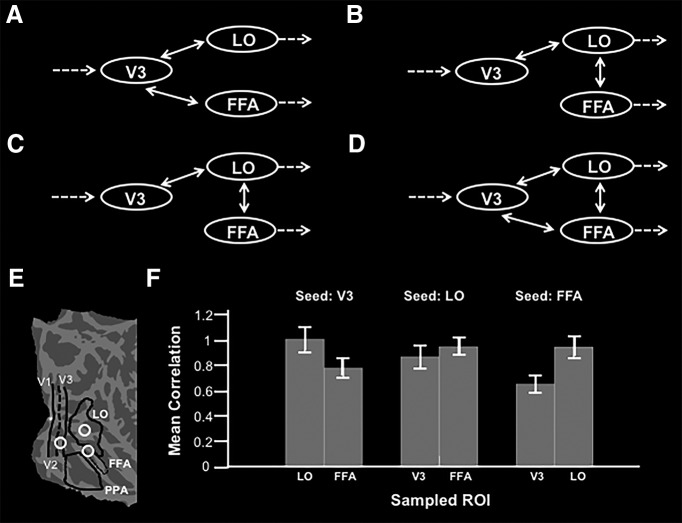
Functional connectivity analysis methods and results from Experiment 4. ***A***, Possible organization for LO and FFA with V3 as a common input. ***B***, ***C***, Alternative possibilities for a hierarchical architecture between LO and FFA. ***D***, Hybrid alternative. ***E***, Flattened-view format of the right hemisphere of a common surface showing the regions of the cortex used as both seeds and sampling ROIs. ***F***, Mean correlation ± SEM within each of the sampled ROIs for the different seeding configurations for 29 subjects.

In all three experiments above, both LO and FFA showed comparable stimulus preferences. Conceivably, this response covariation could reflect a neural link between these two areas (e.g., arising from common inputs from earlier visual areas; Fig. 11*A*), from a hierarchical relationship between LO and FFA (Fig. 11*B*,*C*) or from a combination of both (Fig. 11*D*).

To test for such links, we measured functional connections based on resting-state fMRI in subjects with eyes closed (*n* = 29). Functional connectivity strength was measured among LO, FFA, and an equidistant region in V3 (see Materials and Methods). By limiting functional connectivity measurements to a region of early visual cortex equidistant to both LO and FFA, it became feasible to minimize the alternative possibility that a strong correlation in activity between LO and FFA arose simply because these areas lie close to each other in the cortical map (Salvador et al., 2005). Such a proximity bias is also prominent in studies using neuroanatomical connections in animals (Vezoli et al., 2004; Markov et al., 2011).

Three analyses were performed with these ROIs. For each analysis, one ROI served as a seed while the correlation strength was sampled from the remaining two ROIs. The results were as follows. First, a *post hoc t* test showed that the seed in V3 had a higher connectivity strength with LO compared with FFA^z^ (*p* < 0.10^−3^; *t*_(28)_ = 4.10). Next, a *post hoc t* test showed that the seed in LO had equal connectivity strength with V3 and FFA^aa^ (*p* = 0.22; *t*_(28)_ = −1.26. Finally, a *post hoc t* test showed that the seed in FFA had a higher connectivity strength with LO, compared with V3^bb^ (*p* < 10^−5^; *t*_(28)_ = 5.88). Together, these results indicate that functional connectivity from FFA is more selective for LO than vice versa (see Discussion).

## Discussion

### Neural organization of the smooth bias

Our hypothesis tested whether the responses generated in LO by the contrast of intact and scrambled objects results from low-level feature differences between these image sets, at least in part. This hypothesis, combined with results from Experiments 2a, predicted that the pattern of activity observed for the contrast of smooth versus textured stimuli should resemble the pattern of activity evoked by the contrast of intact versus scrambled objects.

Our results support this hypothesis. We found that the balance of visual responses to smooth versus textured surfaces changed progressively across visual cortical areas, akin to the responses to the contrast of intact versus scrambled objects (Fig. 8*A*,*B*; Malach et al., 1995; Grill-Spector et al., 1998a,b).

This change in response is approximately in accord with the cortical hierarchy, as extrapolated from macaque (Desimone et al., 1985; Felleman and Van Essen, 1991) to humans. Specifically, we found that areas V1, V2, and V3 responded more to textured stimuli compared with smooth stimuli, whereas LO and FFA responded with the reversed bias. Generally, this response pattern paralleled the pattern found for the contrast between intact and scrambled objects, as follows: areas V1, V2, and V3 had a higher response to the relatively more textured (scrambled) state of the objects, whereas LO and FFA showed a higher response for the smoother (intact) state. Moreover, in both experiments, the response bias was strongest in V1 and decreased progressively in V2 to V3, which is also consistent with the cortical hierarchy of these areas in macaque (Desimone et al., 1985; Felleman and Van Essen, 1991).

The smooth and textured labels were chosen to provide an intuition of the difference between high-index and low-index stimuli. Other dimensions may also differentiate the stimuli we used (see below). Nevertheless, the appropriateness of these labels was supported by behavioral data from naive subjects. Presumably, further research can clarify which image statistics, and/or which perceptual dimension, best clarifies the underlying cortical processing.

### Category selectivity

It can be argued that the smooth versus textured distinction comprises an elementary category selectivity, albeit one characterized by midlevel features rather than semantic relationships. Such a feature-based category selectivity would be conceptually intermediate between (1) the higher-level semantic category selectivity reported in FFA, PPA, and extrastriate body area (EBA; i.e., faces, places, and body parts), and (2) the feature-based stimulus selectivity in V1, V2, and MT (e.g., orientation, direction, and color). In other words, the moderately complex distinction of smooth versus textured stimuli observed within LO fits well with the characterization of LO as a middle-tier visual area.

Alternatively, the observed selectivity for smooth versus textured stimuli can be interpreted as a distinction between subordinate categories, which can be distinguished from the ordinate categories that reportedly underlie neural response variations in higher-tier areas, such as FFA, PPA, and EBA (Kanwisher et al., 1997; Epstein and Kanwisher, 1998; Downing et al., 2001; Kanwisher and Yovel, 2006). Subordinate categories depend on ordinate categories (i.e., there must be an ordinate category of “object” in order to define the subordinate categories “smooth objects” and “textured objects”). On the other hand, feature-based categories can be defined without more general semantic categories (i.e., visually smooth and textured surfaces can be distinguished from each other without the more general concept of object). Future work may distinguish between these two interpretations.

Ostensibly, our evidence that FFA responds selectively to this feature-based category of smooth surfaces appears to conflict with a previous conclusion, that FFA responds to the semantic category of faces (Kanwisher et al., 1997; Grill-Spector et al., 2004). However, these differing conclusions are not incompatible, to the extent that amplitude-based signals in FFA reflect both axonal firing and subthreshold neural summation (Logothetis et al., 2001; i.e., possible ascending or descending inputs, and/or intrinsic processing).

### Basis of the bias for smooth objects

The observed bias for smooth surfaces in LO and FFA may seem counterintuitive, because textured surfaces have relatively more local contrast variation compared with smooth surfaces. Thus, to the extent that the visual system responds to the level of local contrast in a given image, the textured stimuli (not the smooth stimuli) should produce relatively higher activity. At lower cortical levels, that result was in fact observed: early (retinotopic) visual areas all showed relatively higher responses to textured stimuli, highest in V1, and decreasing progressively in V2 and V3, in accord with the increasing level of cortical hierarchy. In presumptively higher levels, the converse preference for smooth stimuli in LO and FFA was evident. Overall, the evidence suggests a systematic change in the nature of stimulus processing with increases in hierarchical level (Felleman and Van Essen, 1991; Grill-Spector and Malach, 2004) and mean receptive field size (Yoshor et al., 2007; Dumoulin and Wandell, 2008; Kay et al., 2013).

Although the present study takes into account numerous image features that distinguish between smooth and textured stimuli, it is ultimately possible that the observed effects can be explained by a single factor (e.g., spatial frequency or fractal dimension). However, testing all such hypotheses would require a parametric explosion (i.e., systematic testing of each of the factors that differ between intact and scrambled objects), and thus this question is beyond the scope of the current study. However, our results are unlikely to be explained by factors as simple as spatial frequency content, at least for LO. Past studies have reported a response bias in LO for the high-spatial frequency content of objects and faces (Goffaux et al., 2011; Canário et al., 2016), which would predict a higher response to textured stimuli, contrary to our results.

We can only speculate why the observed bias for smooth surfaces arises. One possibility is that the observed bias arises from an interaction between (1) the roles of LO and FFA in the extraction of 3D shape (Cant and Goodale, 2007, 2011; Georgieva et al., 2008), and (2) large-scale surface-related shape cues (e.g., shading and specular reflection) that are more evident on the surfaces of smooth objects compared with textured objects. This possibility is supported by psychophysical studies showing that material properties can influence the perception of 3D shape (Fleming et al., 2004; Ho et al., 2008). Moreover, the addition of texture to an otherwise smooth surface reduces human performance in the recovery of 3D shape (Cavanagh, 1987; Johnston and Passmore, 1994; Wijntjes et al., 2012).

It is worth noting that textured surfaces can arise in different ways. For example, textures can reflect differences in intrinsic pigmentation or a thin smooth overlay (e.g., paint). Alternatively, variations in surface texture can arise from 3D nonuniformities (i.e., physically “rough” surfaces), combined with nonuniform lighting, on a surface that is undifferentiated by pigment variations. Further experiments may clarify the observed smooth surface bias.

One group has reported that attention to stimulus form versus texture modulates activity in LO and PPA, but not FFA (Cant and Goodale 2007, 2011). In contrast, results here suggest that (bottom- up) stimulus processing (with attention directed elsewhere) shows sensitivity to a surface-versus-texture dimension, in both LO and FFA, without a consistent effect in PPA. The differences in stimulus design (e.g., varying either the locus of attention or the stimuli) and results (e.g., PPA vs FFA) make direct comparisons difficult. Further experiments are required.

### Texture synthesis model

The model used here to measure and synthetize stimuli was originally developed to describe the structure found within visual textures as completely as possible. That model was used here because of the large number of low-level features it considers in the parametric description of an image. This was crucial for our goal of characterizing how features change within an image as an object is grid scrambled (i.e., in the common control condition used to test for object selectivity).

The performance of this model in capturing the structure of visual textures has been validated psychophysically by showing that the multiple sets of parameters the model takes into account are required to achieve near-chance performance in an oddball detection task (Balas, 2006). Here, we extended the validity of this model by showing that naive subjects could classify the appearance of visual objects well above chance, when these objects differed significantly in the measured features (Experiment 2b).

The limits of this model in describing an image are exemplified by our results from Experiment 1a. Subjects performed at chance when asked to identify TS stimuli that were matched in all measured features to the original intact objects. This suggests that the visual system captures more features of the image than the model takes into account. Development of more sophisticated models that capture a more comprehensive set of features could help to extend the results presented here.

### Low-level influences on LO activity

The grid-scrambling process used to generate the control stimuli for the standard LO localizer is designed to render intact objects unrecognizable and/or strip them of their “object-ness” while preserving the pixel values within an image. By comparison, the stronger responses to intact objects in LO are interpreted as being object selective (Grill-Spector et al., 1998b; Kourtzi and Kanwisher 2000). Such interpretations are adopted even when the localization is based on passive viewing (Malach et al., 1995; Grill-Spector et al., 1998b), in which recognition or interpretation of object-ness is not required.

Especially in such passive viewing conditions, LO may be responding, at least in part, to the prominent low-level differences that distinguish the intact and grid-scrambled states of the objects, independent of any differences in recognition or object-ness. Relative to the images of intact objects, grid scrambling introduces rectilinear edges of varying contrast (locally darker or lighter) throughout the original object image. Furthermore, any edges that are present in the original object images are distorted, shortened, and redistributed within the scrambled image. Purely in terms of their image properties, the scrambled objects are quite different from the original unscrambled images. Thus, the classic intact-versus-scrambled LO localizer manipulates both lower-level and higher-level image variables.

The relative balance of these lower-order versus higher-order influences on LO activity is addressed elsewhere (Malach et al., 1995; Grill-Spector et al., 1998a,b,2000; Larsson and Heeger, 2006; Hemond et al., 2007; Scholte et al., 2013; Rice et al., 2014), and we do not attempt to fully resolve that controversy here. Our current experiments largely reflect the influence of low-level features in LO, partly by design. We found that the amplitude of LO responses was only 30% lower to unfamiliar nonobjects with partially matched low-level features (i.e., TS stimuli; Table 3), compared with the responses to intact familiar objects. In prior studies, the reduction in amplitude to nonobjects compared with objects is comparable, typically between 25% and 50% (Grill-Spector et al., 1998a,b). Moreover, any remaining response discrepancy might simply reflect the influence of the residual low-level feature differences between the objects and their TS counterparts, as described above.

This prominence of low-level influences in our current data may also reflect the nature of the dot detection task that subjects performed during the acquisition of fMRI data. This dummy attention task diverts the subjects’ attention from the object stimuli, which reduces the influence of recognition-related processes on the measured response (Guggenmos et al., 2015). Thus, presumably our measurements more prominently reflect low-level cues, by exclusion. By the same token, our results do not rule out possible additional modulation by stimulus type (e.g., objects vs nonobjects) or recognition performance, in other experimental contexts. In any event, our results indicate that activity in LO can be strongly modulated by the measured image features that are manifest in smooth versus textured surfaces.

### Neural relationship between LO and FFA

Several lines of evidence suggest that the smooth bias in LO reflects an intermediary step in the processing of objects and other visual stimuli. First, human area LO has been suggested to occupy a middle tier in the human cortical processing hierarchy area (Malach et al., 1995; Grill-Spector and Malach, 2001; Malach et al., 2002; Gauthier et al., 2012), and this idea is also consistent with the location of LO in the cortical map. Specifically, (1) LO is located between human areas V4 and MT+ (Tootell et al., 1996); (2) in macaque monkeys, V4 and MT are both middle-tier areas (Markov et al., 2014) ; and (3) cortically adjacent areas are often, though not always, strongly interconnected (Vezoli et al., 2004; Markov et al., 2011).

In the present experiments, we found that LO and FFA shared a response bias for smooth surfaces in all three stimulus-driven experiments. Due to well known limitations of noninvasive techniques in humans, we can only speculate about possible circuitry that may underlie the smooth bias in LO and FFA. Several (but not all) possible simplified circuits are diagrammed in Figure 11. Of course, multiple additional connections (not included in the diagrams) almost certainly exist.

We measured functional connectivity among LO, FFA, and V3 to discriminate between the various possible circuit architectures, albeit tentatively. Our results showed that FFA seeds had a particularly high connectivity strength with LO, but the reverse seeding had less specificity. These results are inconsistent with the architectures depicted in Figure 11, *A* and *C*. The former architecture would predict an identical connectivity strength with V3 for LO and FFA, and the latter would predict a stronger connectivity strength between V3 and FFA compared with LO. However, this technique does not allow us to discriminate between the remaining possibilities (Fig. 11*B*,*D*), which both posit direct connections between LO and FFA. This direct relationship between LO and FFA is supported by a recent neuroanatomical study in macaque, which traced the input connections of face-processing network (Grimaldi et al., 2016). That study reports that face-selective regions receive input from non-face-selective regions in temporal occipital area (TEO), including the macaque homolog of LO (Tsao et al., 2003; Orban et al., 2004). In humans, dynamic casual modeling of functional connectivity data also supports the idea of information flow from LO to FFA (Nagy et al., 2012). It is possible that direct connections between LO and FFA are responsible for the shared response bias.

On the other hand, other alternative connections could mediate at least some of our results. The connectivity we found between FFA and early visual cortex (though weak) could support information flow between these cortical regions. Indeed, additional evidence suggests the existence of direct connections between early visual cortex and FFA. In macaques, there is evidence for feedforward anatomical connections from V2, V3, and V4 with the face-processing network (Grimaldi et al., 2016). In humans, MR diffusion imaging suggests anatomical connections between early visual cortex and FFA (Gschwind et al., 2012). Moreover, the finding that patients with object agnosia due to lesions in LO are unimpaired in face recognition abilities suggests that face-selective regions receive input independent from LO (Moscovitch et al., 1997). Further research will be required to resolve these questions.

The hypothetical link between LO and FFA is reminiscent of several discrete streams that are well established in lower levels of visual cortex. For instance, the strong orientation selectivity that is well known in V1 is preserved in orientation-selective regions of V2 (Hubel and Livingstone 1987; Gegenfurtner et al., 1996; Vanduffel et al., 2002), V3 (Gegenfurtner et al., 1997; Vanduffel et al., 2002), and V4 (Tanigawa et al., 2010). Similarly, the direction selectivity that is evident in some V1 cells (especially in layer 4B) presumably forms the functional basis for the prominent direction selectivity that is also reported in V2 (Shipp and Zeki, 2002; Lu et al., 2010), V4 (Li et al., 2013), MT (Dubner and Zeki, 1971; Albright 1984; Movshon and Newsome, 1996), and MST (Saito et al., 1986; Duffy and Wurtz, 1991). In all these examples, selectivity for a given stimulus feature is observed in one initial area and is preserved in subsequent downstream areas.

## Synthesis

The decision was a result of the Reviewing Editor Leonard Maler and the peer reviewers coming together and discussing their recommendations until a consensus was reached. A fact-based synthesis statement explaining their decision and outlining what is needed to prepare a revision is listed below. The following reviewer(s) agreed to reveal their identity: Dwight Kravitz

The two reviews are largely in alignment. Both reviewers agree that the experiments have produced interesting data and that this Ms is potentially publishable in eNeuro.

However, there are many specific concerns. The methods are tricky and the analyses

not transparently related to the questions at hand. The manuscript needs to be framed more clearly and revisions are needed in all sections of the text.

The authors will also need to address the specific concerns of both reviews.

Below please find, in their entirety, both reviews. If you do re-submit to eNeuro, please address all the points raised by both reviewers.

Reviewer 1:

This study uses fMRI to explore the selectivity of visual cortex. The specific aim is to compare the responses to objects with smooth compared to textured surfaces. I think this is a potentially interesting study and I am sympathetic to the approach. However, it took me a number of attempts to understand what was done. Basically, it is not easy to read. So, in its current form it is not suitable for publication.

Experiment 1 uses the Portilla and Simoncelli (2000) algorithms to generate synthetic stimuli based on either intact or scrambled objects. The authors show that it is not possible to recognize the synthetic stimuli. Nonetheless, they show that LO responds more to TS (synthetic from intact) compared to TS- (synthetic from scrambled). This is an interesting finding because it shows that the selectivity for intact vs scrambled is not fully explained by object vs non-object. However, I think that using a ratio is misleading as it can artificially inflate values, particularly if the denominator is small. A difference value or (TS - TS-) / (TS + TS-) would be better ways of showing selectivity. I would also prefer to see the raw data for intact, TS and TS- for each ROI. I know this is in the table, but I think this is important enough to be in a figure.

Experiment 2 uses a data driven approach to look for objects, whose intact and scrambled versions have similar or dissimilar image properties (as defined by the Portilla and Simoncelli (2000) algorithm). This generates 16 similar objects and 16 dissimilar objects. The authors describe these as 'non-preferred' and 'preferred' objects respectively. This is confusing. They should try and use labels that reflect how they were defined rather than the expected response in a particular brain region. The authors then noticed that they were very different. The similar objects had a complex (high spatial-frequency) texture, and the dissimilar objects had a less complex (low spatial-frequency) texture. I think this is a nice finding. However, the authors then renamed the objects as 'smooth' or 'textured'. This is confusing, as this was not how the objects were chosen. It is clear from the objects that changes in luminance across the 'smooth' objects reflect the overall shape (from shading) of the object more than for the 'textured' objects. Perhaps, it is this shape information that is conveyed by these objects that is important. This would change the interpretation of the study. Again, I would prefer to see the raw data from each ROI plotted (see above).

Data from the PPA is conspicuous by its absence. The authors define the region in the figures but don't report the data in any of the figures or the results. The authors should report this data, particularly in light of the published work by Cant and Goodale. This work should also be integrated into the introduction. The main conclusion from this work is that it shows selectivity for shape (LO + FFA) and selectivity for texture (PPA). This doesn't seem too different from the current study, except that the shape is replaced by smooth.

Overall, I think this is an interesting study. However, the text needs to be revised radically if it is to be acceptable for publication.

Minor Points

How were the faces and scenes matched retinotopically?

I think it would be helpful to expand on the way early visual areas were defined. The current definition provides a fairly limited account

Early visual areas V1, V2, and V3 were defined by contrasting the activity maxima to horizontal and vertical meridian stimuli, as described above.

Reviewer 2

In the manuscript the authors report a stronger response in LO and FFA to smooth than textured objects and faces, while the early visual areas show the opposite bias. On this basis they argue that "Thus, LO and FFA may include an information-processing stream distinguished by feature-based category selectivity (smooth > textured)."

The manuscript is well written, the methods clear, and the presentation of the data largely fine. My primary concern is the framing of the result (see below). To my mind, the most interesting thing about this finding is the different biases in LO and FFA and the early visual areas. I wished that the authors had organized the paper around this distinction and provided a more thorough interpretation of the results. Why could underlie this smooth selectivity? What are it implications? The interpretation is now given relatively short shrift. Nonetheless, this should be easily fixed/addressed which will strengthen the manuscript.

LO → FFA Hierarchy

In the last paragraph of the introduction and in the discussion more prominently, the authors raise the idea that LO is somehow a lower level area feeding into FFA. I know of no evidence for this whatsoever, and the ability to find dissociations between prosopagnosia and object agnosia would seem to argue against it. The two regions are equally distant from retinotopic cortex, are biased towards different portions of the visual field, and LO doesn't feature in any models of the face network. I understand that the authors may believe in this relationship, but there is no basis for the assertion in the literature and scant evidence to support it here. Even if it were true, why would LO also provide input to PPA? If LO really does low level shape analysis, wouldn't it feed into PPA as well? Further, it seems sort of orthogonal to the main point of the paper anyway. I would remove it and focus on the issues and evidence relating to the smooth vs. textured result. The fact that FFA shares this bias is not so mysterious that it requires such a complex explanation. Both proximity to LO and the prototypical smoothness of faces are simpler explanations that a hierarchical relationship between the two.

Functional Connectivity Analysis

The functional connectivity analysis is interesting but there are a number of aspects to it that are confusing. First, why were the FC ROIs defined on the common surface? Why not perform this analysis for each participant in his or her native space? Second, the analysis itself, while very clever, seems fraught with unaddressed issues. For example, the top two panels suggest that the analysis will differentiate between a direct connection between V2/V3 and FFA or one that comes through LO. However, the flat maps don't even show the position of V2/V3 and their center is never used as a seed in the analysis. If it were, it would drive home how odd it is to use the distances between two ROIs as a constraint for reasoning about a third. Why not just show the connectivity maps that result from each of three seeds placed in the middle of each ROI? Im not sure how else to rescue this analysis, but Im also not clear what it adds to the paper (see above).

For all flat maps it is necessary to indicate the thresholds that were used to define each map.

If PPA features in the flat maps and the interpretation so heavily, then its smooth/freckled ratio should also be shown in the bar plots.

All 32 of the smooth and textured stimuli should be shown in the figure.

It would have been nice to include real and scrambled stimuli in Experiment 1c so that their ratios could have been quantified relative to the TS and TS-. There is no need to collected further data, but these ratios are in the literature and should be reported here for comparisons sake (pg 16).

The t-test on the bottom of page 17, establishing the difference between the two conditions in the position indices is entirely circular and should be removed. The two conditions were chosen to have different position indices. No test is necessary or appropriate.

The overlap in participants from the pool of 30 across experiments should be described in the methods. 37/30 participated in experiments 1-3.

The data in figure 6A is described for the intact objects but not for the other conditions. The data is in the figure, so either describe it in the text for all conditions or just let the figure carry all of it.
